# The “fruit and whole‐grain” pattern is associated with a low prevalence of hypertriglyceridemia among middle and older‐aged Korean adults: Using Korea National Health and Nutrition Examination Survey 2013–2018 data

**DOI:** 10.1002/fsn3.3128

**Published:** 2022-11-04

**Authors:** SoHyun Park, Sangwon Chung, Seong‐Ah Kim, Sangah Shin

**Affiliations:** ^1^ Department of Food and Nutrition Chung‐Ang University Gyeonggi‐do Korea; ^2^ Personalized Diet Research Group Korea Food Research Institute Jeollabuk‐do Korea; ^3^ Department of Urban Society Research The Seoul Institute Seoul Korea

**Keywords:** dietary pattern, fruit, hypertriglyceridemia, KNHANES, whole‐grain

## Abstract

Hypertriglyceridemia is a well‐known risk factor of various chronic diseases including diabetes mellitus, metabolic syndrome, obesity, and cardiovascular diseases. This study aimed to determine dietary patterns and explore the relationship between dietary patterns and hypertriglyceridemia in the Korean adult population. We utilized a cross‐sectional and nationally representative survey, the Korea National Health and Nutrition Examination Survey 2013–2018 database. From 47,217 subjects who participated in the survey between 2013 and 2018, only subjects over 40 years old were included. Subjects lacking 24‐h recall data and data on hypertriglyceridemia and body mass index, and who had implausible energy intake were excluded. A total of 19,806 participants' data were analyzed. Dietary data were based on 24‐h recall data, and dietary patterns were derived using factor analysis. Triglyceride levels greater than 200 mg/dl were considered hypertriglyceridemia, according to the Korean Society of Lipid and Atherosclerosis. Three dietary patterns— “oil and fats & seasoning”, “soybean paste and vegetable”, and “fruit and whole‐grain”— explained 7.9%, 6.3%, and 5.8% of variation in food intake, respectively. Comparing the lowest and highest dietary pattern score groups after adjusting for potential confounders revealed an inverse relationship between “fruit and whole‐grain” dietary pattern and hypertriglyceridemia in men (odds ratio [OR]: 0.61, 95% confidence interval [CI]: 0.45–0.82, *p* for trend <.0001); which was only marginal in women (OR: 0.78, 95% CI: 0.58–1.07, *p* for trend: .628). A diet containing high proportions of fruit and whole‐grain may have preventive effects on hypertriglyceridemia in middle and older aged Korean adults.

## Graphical abstract

This study aimed to derive dietary patterns and explore the relationship between dietary patterns and hypertriglyceridemia in the Korean middle and older aged adult population using data from a representative national survey in Korea. As a result, an inverse relationship between “fruit and whole‐grain” dietary pattern and hypertriglyceridemia was observed in men. A diet containing high proportions of fruit and whole‐grain may prevent hypertriglyceridemia in Korean adults.

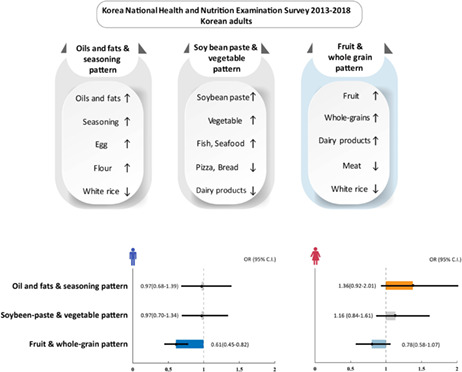

## 1 INTRODUCTION

Hypertriglyceridemia, an elevation in triglyceride (TG) levels, is an important biomarker of cardiovascular disease (Brunzell, 2007). Consistently high TG levels may lead to changes in the composition and metabolism of low‐ and high‐density lipoprotein (LDL and HDL) cholesterol, thereby causing an imbalance in serum cholesterol levels (Miller et al., 2011). Moreover, hypertriglyceridemia in conjunction with impaired fasting glucose is a well‐known risk of the development of type 2 diabetes (D'Agostino et al., 2004). Since hypertriglyceridemia may affect other metabolic factors, it is currently considered a major risk factor for various chronic diseases.

For a long time, dietary control has been advocated for managing hypertriglyceridemia. Studies have examined the association between dietary factors and hypertriglyceridemia. According to a previous study, the TG level was expected to increase by 6% for every 5% decrease in the percent of total fat intake in those who consumed a low‐fat and high‐carbohydrate diet (Institute of Medicine, 2005). Furthermore, studies have reported a link between the consumption of several food items and the development of hypertriglyceridemia (Guasch‐Ferre et al., 2019; Takahashi et al., 2010; Yuan et al., 2015). The consumption of more than three servings of fruits and vegetables and whole grains led to a reduction in TG levels in Brazilian adults (Takahashi et al., 2010). Additionally, a high intake of fruit, but not vegetables, was related to a decrease in the prevalence of elevated TG levels in Korean adults (Yuan et al., 2015). A meta‐analysis of randomized controlled trials showed that the consumption of red meat resulted in a greater decrease in the level of TG than that of low‐quality refined grains and simple sugars (Guasch‐Ferre et al., 2019).

The dietary pattern analysis can be used as an adjunct method to explore the combined effect of nutrients and foods in a particular population and examine the relationship between overall diet and risk of metabolic diseases (Hu, 2002). In this regard, the association of dietary patterns with hypertriglyceridemia has been evaluated. A healthy dietary pattern, characterized by high consumption of whole grains, legumes, fruits, and vegetables, was inversely associated with low levels of TG in the Western population (Panagiotakos et al., 2007). Moreover, a similar dietary pattern was negatively associated with fasting TG in a multiethnic Asian population (Whitton et al., 2018).

Importantly, the relationship between specific dietary patterns and triglycerides has not been clearly elucidated in the Korean population. In a previous study involving Korean adults, a healthy dietary pattern, including grains, vegetables, and fish, was associated with a lower risk of hypertriglyceridemia than a traditional dietary pattern (Kim & Jo, 2011), while in another similar study, it showed a nonsignificant relationship (Song & Joung, 2012). Therefore, we aimed to derive dietary patterns and explore the relationship between dietary patterns and hypertriglyceridemia in the Korean adult population using data from a representative national survey in Korea.

## 2 EXPERIMENTAL SECTION

### 2.1 Study population

**FIGURE 1:**
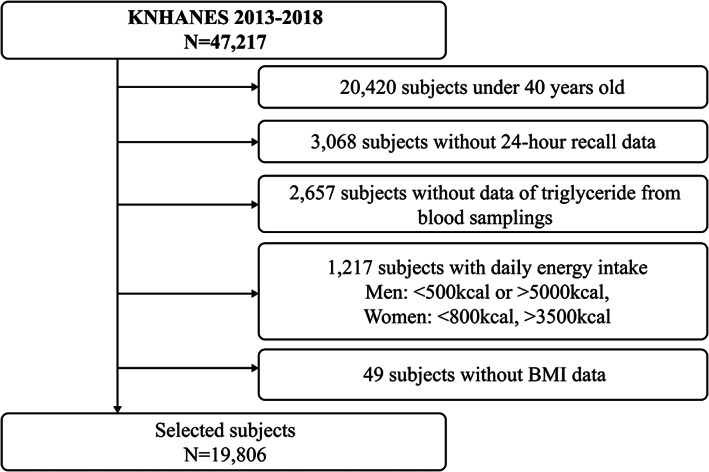
Flow diagram of analytical samples in this study: Korea National Health and Nutrition Examination Survey 2013–2018

In this study, we utilized the database of the Korea National Health and Nutrition Examination Survey (KNHANES)—a cross‐sectional and nationally representative survey conducted by the Division of Chronic Disease Surveillance, The Korea Disease Control and Prevention Agency. The KNHANES was approved by the Institutional Review Board of the Korea Disease Control and Prevention Agency (2013‐07CON‐03‐4C, 2013‐12EXP‐03‐5C, and 2018‐01‐03‐P‐A). Among the 47,217 subjects who participated in the survey between 2013 and 2018, subjects who were less than 40 years old (*n* = 20,420) and lacked 24‐h recall data (*n* = 3068), data on hypertriglyceridemia (*n* = 2657) and body mass index (BMI) (*n* = 49) were excluded. In addition, 1217 subjects who had implausibly high (male, >5000 kcal/day; female, >3500 kcal/day) or low (male, <500 kcal/day; female, <800 kcal/day) energy intake were excluded. Finally, a total of 19,806 participants' data were included in the analysis (Figure 1).

### 2.2 Dietary intake and dietary pattern

Dietary intake and the dietary patterns were based on the 24‐h recall data. Data on intake of major macronutrients, carbohydrate, protein, and fat were obtained and percentage energy from these macronutrient intakes was assessed as follows: macronutrient intake (g/day) * kcal/g (carbohydrate and protein: 4 kcal/g, fat: 9 kcal/g)/total energy intake (kcal/day). The food items were categorized into 24 food groups based on the similarity of food type and nutrient composition. The specific food components of each food group are described in Table S1. Using factor analysis, different dietary patterns were identified from daily food intake within each food group. Principal component analysis was conducted to identify the distinct dietary patterns. The number of factors was determined using eigenvalues >1.3 and scree plots. The factor score for each dietary pattern was calculated through the sum of food intake weighted by their factor loadings. Three factors with high positive loadings were used to derive and label dietary patterns: “oil and fats & seasoning,” “soybean paste and vegetable,” and “fruit and whole‐grain.” The subjects were divided into three groups based on their dietary pattern scores.

### 2.3 Hypertriglyceridemia

Hypertriglyceridemia was defined if triglyceride level of participants is greater than 200 mg/dl, based on the guidelines of the Korean Society of Lipid and Atherosclerosis (Committee for the Korean Guidelines for the Management of Dyslipidemia, 2016), or participants are taking medication for lowering blood lipids.

### 2.4 Covariates

Sociodemographic and health‐related behavior data, including household income level, physical activity, alcohol consumption, and smoking were obtained via a self‐administered questionnaire. Household income level was classified as low, medium‐low, medium‐high, and high. Physical activity status was defined according to the frequency of walking and categorized as none, <3 days/week, and 4–7 days/week. Current alcohol consumption was determined as the presence or absence of alcohol intake in the recent 1 year (“yes” and “no”). Based on the smoking status, the subjects were categorized as “non‐smokers” and “smokers.” Height and weight were measured using standard protocol by trained staff. BMI was calculated as the weight divided by the square of the height (kg/m^2^).

### 2.5 Statistical analysis

Statistical analyses were performed using SAS software (version 9.4; SAS Institute, Inc.). Statistical significance was set at *p* < .05. Categorical data were presented as numbers and percentages and continuous data as means with standard error. The differences across quantiles of dietary pattern scores were determined using the generalized linear model for continuous data and chi‐square test for categorical data. Multivariable‐adjusted logistic regression analysis was used to calculate the odds ratio (OR) and 95% confidence interval (CI) for hypertriglyceridemia.

Model 1 was adjusted for age (continuous) and other dietary pattern scores (continuous). Model 2 was additionally adjusted for total energy intake (continuous), household income (low, middle‐low, middle‐high, high), current smoking status (yes, no), alcohol consumption status (yes, no), and physical activities (none, <3 days/week, and 4–7 days/week). Model 3 was additionally adjusted for energy‐adjusted carbohydrate (continuous) using the residual method (Willett & Stampfer, 1986).

## 3 RESULTS

The factor loadings for three dietary patterns are shown in Table 1. The “oil and fats & seasoning” pattern included high consumption of oil and fats, seasoning, egg, flour, and low consumption of white rice. The “soybean paste and vegetable” pattern included high consumption of soybean paste, vegetables, fish and seafood, kimchi, and low consumption of pizza, hamburger, bread, sandwich, cereal, and dairy products. The “fruit and whole‐grain” pattern included high consumption of fruit, whole grains, dairy products, potato, and low consumption of meat, alcoholic beverages, and white rice.

**TABLE 1:** Factor loadings^a^ for the major dietary patterns identified according to a factor analysis among middle and older aged Koreans, the 2013–2018 KNHANES

	Dietary patterns		
	Factor 1	Factor 2	Factor 3
	Oil and fats & seasoning pattern	Soybean paste and vegetable pattern	Fruit and whole‐grain pattern
White rice	−0.158	0.453	−0.457
Whole grains		0.217	0.468
Flours	0.366		
Noodles	0.261		
Bread, sandwiches, and cereal		−0.239	
Pizza and hamburgers	0.318	−0.208	
Potatoes			0.372
Fruits			0.487
Vegetables		0.524	0.265
Kimchi		0.361	
Mushrooms	0.165		
Legumes and soybean products		0.292	
Soybean paste		0.575	
Salted foods	0.249		
Eggs	0.396		
Meats	0.330		−0.248
Fish and seafood		0.398	0.174
Dairy products		−0.162	0.416
Beverages	0.359		
Alcoholic beverages	0.265		−0.339
Sugar and syrup	0.317		
Nuts			0.295
Oils and fats	0.673		
Seasonings	0.488		
The variance of intake explained (%)	7.9	6.3	5.8

Abbreviation: KNHANES: Korea National Health and Nutrition Examination Survey.

^a^ Factor loading values of <|0.150| were not listed on the table for simplicity.

The general characteristics of this study population according to quantiles of three pattern scores are described in Table 2. The “oil and fats & seasoning” dietary pattern was positively associated with men who were younger, more active, and had higher alcohol intake, and women who were younger and smoked and consumed alcohol frequently. The “soybean‐paste and vegetable” dietary pattern group included men who were old and tended to drink and smoke often and women who were old and smoked and consumed alcohol infrequently. Likewise, the “fruit and whole‐grain” dietary pattern group included men in a high‐income household who also smoked and consumed alcohol infrequently and women who were young, active, and smoked and consumed alcohol infrequently.

**TABLE 2:** General characteristics according to quantiles of three dietary pattern scores among middle and older aged Koreans, the 2013–2018 KNHANES

	Quantile (Q) of the dietary pattern				*p*‐Value^a^
	Q1	Q2	Q3	Q4	
Men (*N* = 7933)	1983	1983	1984	1983	
Oil and fats & seasoning pattern					
Triglyceride range (mg/dl)	83–178	88–190	89–193	94–202	
Age (year)	61.2 ± 0.5	57.2 ± 0.4	54.1 ± 0.3	51.4 ± 0.3	<.0001
BMI (kg/m^2^)	23.9 ± 0.1	24.4 ± 0.1	24.2 ± 0.1	24.5 ± 0.1	.1149
Household income (high)	290 (18.1)	474 (27.6)	602 (34.0)	776 (42.5)	<.0001
Physical activity (>3 days/week)	335 (28.8)	407 (29.6)	399 (29.1)	424 (31.6)	<.0001
Current alcohol drinkers	1331 (78.4)	1454 (82.7)	1580 (87.4)	1635 (89.0)	<.0001
Current smokers	1560 (81.5)	1559 (81.3)	1585 (83.8)	1580 (81.7)	<.0001
Soybean‐paste & vegetable pattern					
Age (year)	54.2 ± 0.4	55.8 ± 0.4	55.8 ± 0.4	56.6 ± 0.4	.0379
BMI (kg/m^2^)	24.3 ± 0.1	24.4 ± 0.1	24.2 ± 0.1	24.2 ± 0.1	.3260
Household income (high)	568 (33.5)	517 (29.9)	511 (30.2)	546 (32.7)	<.0001
Physical activity (>3 days/week)	411 (31.0)	384 (28.9)	399 (30.8)	371 (28.7)	<.0001
Current alcohol drinkers	1478 (83.4)	1485 (84.8)	1502 (86.8)	1535 (85.1)	<.0001
Current smokers	1553 (81.5)	1581 (82.2)	1567 (82.3)	1583 (82.4)	<.0001
Fruit and whole‐grain pattern					
Age (year)	54.6 ± 0.4	55.9 ± 0.4	55.4 ± 0.4	56.3 ± 0.4	<.0001
BMI (kg/m^2^)	24.1 ± 0.1	24.2 ± 0.1	24.4 ± 0.1	24.3 ± 0.1	<.0001
Household income (high)	476 (28.1)	445 (26.6)	542 (32.9)	679 (39.2)	<.0001
Physical activity (>3 days/week)	344 (27.2)	359 (26.6)	407 (31.5)	455 (34.7)	<.0001
Current alcohol drinkers	1657 (87.9)	1596 (83.0)	1552 (80.4)	1479 (76.8)	<.0001
Current smokers	1641 (91.4)	1511 (85.9)	1411 (80.4)	1437 (81.6)	<.0001
Women (*N* = 11,873)	2968	2968	2969	2968	
Oil and fats & seasoning pattern					
Age (year)	62.3 ± 0.4	56.9 ± 0.4	54.1 ± 0.3	51.7 ± 0.3	<.0001
BMI (kg/m^2^)	24.3 ± 0.1	23.9 ± 0.1	23.8 ± 0.1	23.5 ± 0.1	<.0001
Household income (high)	352 (13.3)	702 (26.4)	909 (33.3)	1110 (38.8)	<.0001
Physical activity (>3 days/week)	549 (27.5)	675 (33.4)	757 (36.9)	759 (37.8)	<.0001
Current alcohol drinkers	1239 (66.1)	1630 (74.3)	1828 (79.0)	1999 (80.3)	<.0001
Current smokers	188 (6.9)	225 (8.1)	231 (8.6)	242 (8.4)	<.0001
Soybean‐paste & vegetable pattern					
Age (year)	54.1 ± 0.4	56.3 ± 0.4	56.8 ± 0.4	57.0 ± 0.4	.0012
BMI (kg/m^2^)	23.8 ± 0.1	23.8 ± 0.1	23.8 ± 0.1	23.8 ± 0.1	.8551
Household income (high)	950 (34.0)	747 (27.7)	690 (26.0)	686 (26.0)	.0031
Physical activity (>3 days/week)	725 (35.0)	669 (34.4)	668 (33.1)	678 (33.9)	<.0001
Current alcohol drinkers	1815 (77.5)	1659 (75.3)	1638 (75.2)	1584 (74.3)	<.0001
Current smokers	278 (10.2)	212 (7.4)	212 (7.6)	184 (6.8)	<.0001
Fruit and whole‐grain pattern					
Age (year)	56.8 ± 0.4	56.1 ± 0.4	55.7 ± 0.4	55.4 ± 0.3	<.0001
BMI (kg/m^2^)	24.1 ± 0.1	23.8 ± 0.1	23.8 ± 0.1	23.6 ± 0.1	<.0001
Household income (high)	561 (20.7)	710 (27.7)	810 (29.8)	992 (35.8)	<.0001
Physical activity (>3 days/week)	559 (28.1)	637 (31.1)	740 (35.9)	804 (41.6)	<.0001
Current alcohol drinkers	1753 (80.3)	1656 (75.6)	1622 (73.7)	1665 (73.2)	<.0001
Current smokers	317 (11.7)	234 (8.6)	179 (6.4)	156 (5.8)	<.0001

*Note*: Values are presented as mean ± standard error for continuous variables and weighted n (percent) for categorical variables.

Abbreviation: KNHANES: Korea National Health and Nutrition Examination Survey; BMI: body mass index.

^a^ *p*‐Values were obtained from generalized linear model for continuous variables and chi‐square test for categorical variables.

The energy and the macronutrient intake according to three dietary pattern scores are presented in Table 3. The participants following the “oil and fats & seasoning” dietary pattern tended to have high energy, carbohydrate, protein, and fat intake and high percentage of energy from protein and fat intake in both men and women. The participants following the “soybean paste and vegetable” dietary pattern tended to have high energy, carbohydrate, protein, and fat intake, and high percentage of energy from carbohydrate intake in men and women. The participants following the “fruit and whole‐grain” dietary pattern tended to have a high proportion of three main macronutrients, which are carbohydrate, protein, and fat. Men who had a high intake of “fruit and whole‐grain” pattern tended to have low energy while women tended to have high energy.

**TABLE 3:** Energy and macronutrient intake according to quantiles of three dietary pattern score among middle and older aged Koreans, the 2013–2018 KNHANES

	Quantile (Q) of the dietary pattern				*p*‐Value^a^
	Q1	Q2	Q3	Q4	
Men (*N* = 7933)	1983	1983	1984	1983	
Oil and fats & seasoning pattern					
Total energy intake	1748.1 ± 24.1	1944.5 ± 21.9	2195.5 ± 20.8	2538.4 ± 21.8	<.0001
Carbohydrate (g/day)	319.9 ± 4.7	319.3 ± 3.9	338.3 ± 3.9	354.0 ± 4.2	.0011
% energy from carbohydrate	73.2 ± 0.4	66.1 ± 0.4	62.2 ± 0.5	56.3 ± 0.5	<.0001
Protein (g/day)	52.4 ± 0.9	63.7 ± 0.9	74.1 ± 0.9	93.6 ± 1.3	<.0001
% energy from protein	12.0 ± 0.1	13.3 ± 0.2	13.6 ± 0.2	14.8 ± 0.1	<.0001
Fat (g/day)	22.0 ± 0.6	33.4 ± 0.8	42.3 ± 0.8	57.0 ± 1.1	<.0001
% energy from fat	11.1 ± 0.2	15.2 ± 0.3	17.3 ± 0.3	20.2 ± 0.3	<.0001
Soybean paste and vegetable pattern					
Total energy intake	1952.8 ± 26.6	2012.3 ± 24.9	2152.1 ± 21.2	2471.7 ± 22.8	<.0001
Carbohydrate (g/day)	293.1 ± 4.3	316.5 ± 4.1	340.4 ± 3.8	393.0 ± 4.4	.0012
% energy from carbohydrate	61.5 ± 0.6	64.5 ± 0.6	64.5 ± 0.5	64.6 ± 0.5	<.0001
Protein (g/day)	63.9 ± 1.2	65.5 ± 1.1	73.0 ± 1.1	89.7 ± 1.2	<.0001
% energy from protein	13.0 ± 0.2	13.0 ± 0.1	13.6 ± 0.2	14.5 ± 0.1	.1928
Fat (g/day)	41.0 ± 1.0	36.3 ± 1.0	39.7 ± 0.9	43.7 ± 1.1	.0039
% energy from fat	18.0 ± 0.3	15.7 ± 0.3	16.1 ± 0.3	15.5 ± 0.3	.1126
Fruit and whole‐grain pattern					
Total energy intake	2322.3 ± 24.6	1991.1 ± 25.8	2032.1 ± 24.2	2214.6 ± 23.3	<.0001
Carbohydrate (g/day)	330.9 ± 4.2	311.2 ± 3.8	328.7 ± 4.2	368.2 ± 4.2	<.0001
% energy from carbohydrate	58.3 ± 0.6	64.2 ± 0.5	65.5 ± 0.4	67.1 ± 0.4	<.0001
Protein (g/day)	74.4 ± 1.2	67.1 ± 1.2	71.3 ± 1.2	78.0 ± 1.3	<.0001
% energy from protein	12.7 ± 0.1	13.4 ± 0.1	14.0 ± 0.2	14.1 ± 0.2	<.0001
Fat (g/day)	39.4 ± 0.9	36.4 ± 1.1	40.2 ± 0.9	44.9 ± 1.0	<.0001
% energy from fat	14.7 ± 0.3	15.6 ± 0.3	17.3 ± 0.3	17.8 ± 0.3	<.0001
Women (*N* = 11,873)	2968	2968	2969	2968	
Oil and fats & seasoning pattern					
Total energy intake	1388.9 ± 17.2	1499.8 ± 15.6	1644.6 ± 15.7	2093.2 ± 17.1	<.0001
Carbohydrate (g/day)	268.3 ± 3.6	266.2 ± 3.3	272.7 ± 3.2	313.0 ± 3.2	<.0001
% energy from carbohydrate	77.0 ± 0.3	70.6 ± 0.4	66.1 ± 0.3	60.0 ± 0.4	<.0001
Protein (g/day)	39.5 ± 0.5	48.1 ± 0.6	57.1 ± 0.7	79.0 ± 1.0	<.0001
% energy from protein	11.5 ± 0.1	13.0 ± 0.1	14.0 ± 0.1	15.1 ± 0.1	<.0001
Fat (g/day)	16.0 ± 0.4	25.3 ± 0.5	33.1 ± 0.6	52.7 ± 0.9	<.0001
% energy from fat	10.5 ± 0.2	15.3 ± 0.3	18.3 ± 0.3	22.5 ± 0.3	<.0001
Soybean paste and vegetable pattern					
Total energy intake	1482.8 ± 20.9	1529.8 ± 16.8	1684.0 ± 18.0	2012.4 ± 19.7	<.0001
Carbohydrate (g/day)	239.2 ± 3.6	256.2 ± 2.8	288.9 ± 3.0	343.6 ± 3.5	<.0001
% energy from carbohydrate	65.4 ± 0.4	68.3 ± 0.4	69.5 ± 0.4	69.0 ± 0.4	.0497
Protein (g/day)	49.7 ± 1.0	50.3 ± 0.8	56.7 ± 0.8	71.4 ± 1.0	.0053
% energy from protein	13.3 ± 0.2	13.1 ± 0.1	13.4 ± 0.1	14.2 ± 0.1	.2677
Fat (g/day)	33.0 ± 0.8	30.2 ± 0.8	30.4 ± 0.7	37.0 ± 0.8	.0441
% energy from fat	19.2 ± 0.3	16.8 ± 0.3	15.6 ± 0.3	16.0 ± 0.3	.5500
Fruit and whole‐grain pattern					
Total energy intake	1616.4 ± 17.7	1526.0 ± 19.2	1644.4 ± 20.4	1897.7 ± 17.5	<.0001
Carbohydrate (g/day)	268.8 ± 3.1	255.2 ± 3.1	275.5 ± 3.6	323.5 ± 3.6	<.0001
% energy from carbohydrate	67.8 ± 0.5	67.7 ± 0.4	67.9 ± 0.4	68.7 ± 0.4	<.0001
Protein (g/day)	51.7 ± 0.8	51.6 ± 0.8	57.0 ± 0.9	66.8 ± 1.0	<.0001
% energy from protein	12.6 ± 0.1	13.5 ± 0.1	13.8 ± 0.1	14.1 ± 0.2	<.0001
Fat (g/day)	28.2 ± 0.7	29.5 ± 0.8	33.7 ± 0.8	39.0 ± 0.8	<.0001
% energy from fat	14.9 ± 0.3	16.8 ± 0.3	17.9 ± 0.3	18.1 ± 0.3	<.0001

*Note*: Values are presented as mean ± standard error.

Abbreviation: KNHANES: Korea National Health and Nutrition Examination Survey.

^a^ *p*‐Values were obtained from a generalized linear model for continuous variables.

Table 4 shows the ORs and 95% CIs of hypertriglyceridemia across the quantiles of three dietary pattern scores. There were no significant associations between “oil and fats & seasoning” and “soybean paste and vegetable” patterns and the prevalence of hypertriglyceridemia in both men and women. The ORs of the highest quantile (Q4) for the “fruit and whole‐grain” pattern showed a statistically significant association with the prevalence of hypertriglyceridemia in men (OR: 0.61, 95% CI: 0.45–0.82, *p* for trend <.0001; model 3). Likewise, for women participants in Q4, the prevalence of hypertriglyceridemia was significantly decreased in model 1 (OR: 0.68, 95% CI: 0.52–0.90, *p* for trend = .0051) but in model 2 (OR: 0.79, 95% CI: 0.58–1.07 *p* for trend = .0856) and model 3 (OR: 0.78, 95% CI: 0.58–1.07, *p* for trend = .062) it was on the borderline.

**TABLE 4:** The odds ratios of hypertriglyceridemia across the quantiles of three dietary pattern scores among middle and older aged Koreans, the 2013–2018 KNHANES

	Quantile (Q) of the dietary pattern				*p* for trend
	Q1	Q2	Q3	Q4	
Men (*N* = 7933)					
Oil and fats & seasoning pattern					
*N*	1983	1983	1984	1983	
Triglyceride range (mg/dl)	83–178	88–190	89–193	94–202	
Model 1^a^	1.00 (ref.)	0.99 (0.74–1.32)	1.03 (0.77–1.36)	1.12 (0.84–1.48)	.2780
Model 2^b^	1.00	0.95 (0.71–1.28)	1.04 (0.76–1.41)	1.09 (0.77–1.53)	.4864
Model 3^c^	1.00	0.91 (0.67–1.23)	0.96 (0.70–1.32)	0.97 (0.68–1.39)	.9635
Soybean paste and vegetable pattern					
*N*	1983	1983	1983	1984	
Triglyceride range (mg/dl)	90–198	89–191	88–185	88–190	
Model 1	1.00	0.95 (0.72–1.25)	1.01 (0.77–1.32)	0.95 (0.72–1.26)	.9247
Model 2	1.00	0.93 (0.70–1.24)	1.02 (0.761–1.36)	0.93 (0.67–1.28)	.8974
Model 3	1.00	0.95 (0.72–1.26)	1.05 (0.78–1.40)	0.97 (0.70–1.34)	.5518
Fruit and whole‐grain pattern					
*N*	1983	1983	1984	1983	
Triglyceride range (mg/dl)	99–218	90–189	85–182	85–177	
Model 1	1.00	0.72 (0.54–0.95)	0.65 (0.49–0.85)	0.55 (0.41–0.73)	<.0001
Model 2	1.00	0.71 (0.53–0.95)	0.64 (0.47–0.85)	0.56 (0.42–0.75)	.0002
Model 3	1.00	0.73 (0.55–0.99)	0.67 (0.50–0.90)	0.61 (0.45–0.82)	<.0001
Women (*N* = 11,873)					
Oil and fats & seasoning pattern					
*N*	2968	2968	2969	2968	
Triglyceride range (mg/dl)	84–165	78–153	72–147	70–145	
Model 1	1.00	1.10 (0.82–1.48)	1.00 (0.76–1.34)	1.17 (0.88–1.57)	.4361
Model 2	1.00	1.16 (0.85–1.59)	1.08 (0.79–1.46)	1.30 (0.91–1.84)	.2195
Model 3	1.00	1.18 (0.86–1.62)	1.11 (0.80–1.53)	1.36 (0.92–2.01)	.1619
Soybean paste and vegetable pattern					
*N*	2968	2968	2969	2968	
Triglyceride range (mg/dl)	71–147	74–150	78–154	78–159	
Model 1	1.00	0.99 (0.75–1.32)	0.90 (0.67–1.20)	1.10 (0.84–1.45)	.6100
Model 2	1.00	1.06 (0.78–1.42)	0.92 (0.68–1.25)	1.16 (0.84–1.61)	.4598
Model 3	1.00	1.05 (0.78–1.42)	0.92 (0.68–1.24)	1.16 (0.84–1.61)	.6994
Fruit and whole‐grain pattern					
*N*	2968	2968	2969	2968	
Triglyceride range (mg/dl)	76–158	76–160	74–147	73–147	
Model 1	1.00	0.85 (0.65–1.12)	0.71 (0.54–0.92)	0.68 (0.52–0.90)	.0051
Model 2	1.00	0.91 (0.69–1.21)	0.79 (0.60–1.04)	0.79 (0.58–1.07)	.0856
Model 3	1.00	0.91 (0.69–1.21)	0.79 (0.59–1.04)	0.78 (0.58–1.07)	.0628

Abbreviations: KNHANES: Korea National Health and Nutrition Examination Survey, OR: odds ratio, CI: confidence interval.

^a^ Model 1 was adjusted for age (year) and other pattern scores.

^b^ Model 2 was adjusted for model 1+ total energy intake, household income (low; middle‐low; middle‐high; high), current smoking status (yes; no), drinking status (yes; no in recent 1 year), physical activities (no; less than 3 days/week; >3 days/week), and BMI.

^c^ Model 3 was adjusted for model 2 + energy‐adjusted carbohydrate intake.

## 4 DISCUSSION

In this study, using data from 2013–2018 KNHANES, a negative relationship was observed between the “fruit and whole‐grain” dietary pattern and hypertriglyceridemia in middle and older aged Korean adults. Among men following the “fruit and whole‐grain” pattern, the highest dietary pattern score group was significantly associated with a 39% lower prevalence of hypertriglyceridemia than the lowest dietary pattern score group. Among women, there was a marginal negative association between hypertriglyceridemia and “fruit and whole‐grain” dietary pattern.

The assessment of dietary patterns rather than a single nutrient or specific food intake is known to be a better approach to evaluate the association of overall diet with chronic diseases in a population. Dietary guidelines focusing on cardiovascular health, such as dietary approaches to stop hypertension (DASH) and the Mediterranean diet, recommend overall consumption of healthy foods, including fruits, vegetables, whole grains, fish, nuts, legumes, and olive oil (Salehi‐Abargouei et al., 2013; Zazpe et al., 2008). Similarly, the definition of “healthy diet” by the American Heart Association also focuses on whole foods and dietary patterns (Lloyd‐Jones et al., 2010). The relationship between dietary pattern and cardiometabolic risk factors has been assessed previously in various studies. Specifically, healthy dietary patterns, characterized by a high content of vegetables, fish, poultry, fruit, and whole grains, were associated with reduced risk for metabolic syndrome in Asian countries (Fabiani et al., 2019).

As described above, fruit consumption is generally included in healthy dietary patterns to control cardiometabolic parameters (Panagiotakos et al., 2007). Indeed, a high intake of fruits was negatively associated with hypertriglyceridemia in a recent meta‐analysis (Kodama et al., 2018). In addition, increased frequency of fruit consumption (≥3–4 servings/day) was negatively associated with hypertriglyceridemia in the Asian and South American populations (Lim & Kim, 2020; Takahashi et al., 2010). The consumption of total fruit and specific fruit subgroups, such as citrus, noncitrus, and carotene‐rich fruits, also showed an inverse association with hypertriglyceridemia in an observational study (Yuan et al., 2015). In an intervention study, grapefruit supplementation led to a decrease in serum TG in hyperlipidemic patients (Gorinstein et al., 2006).

However, the association between fruit intake and hypertriglyceridemia should be interpreted with caution since a high amount of fructose in fruits may lead to an increase in blood TG levels (Sharma et al., 2016). Dietary fructose led to an increase in postprandial TG levels in the short term, controlled feeding studies (Schaefer et al., 2009), and intake of only vegetables, not fruit, was related to a reduced risk of type 2 diabetes mellitus, which has been linked to hypertriglyceridemia (Colditz et al., 1992; Villegas et al., 2008; Williams et al., 1999), indicating that fruits and vegetables rich in phytochemicals or fibers have an antihypertriglyceridemia effect that is attenuated by fructose (Kodama et al., 2018). Therefore, we evaluated energy‐adjusted carbohydrate intake to exclude the potential confounding effects of simple sugar intake. The results suggesting a favorable effect of fruit intake on hypertriglyceridemia in this study can be useful evidence for a dietary recommendation.

Whole‐grain consumption also plays a potential role in the prevention of hypertriglyceridemia. In a 12‐week nutritional intervention study, a whole grain cereal‐based diet led to a greater decrease in postprandial TG levels than a refined cereal diet (Giacco et al., 2014). In another randomized crossover study, a lower value for incremental area under the curve for TG level was observed in subjects on a whole‐grain diet with dairy, chicken, and nuts than in subjects on a refined grain diet with red meat (Kim et al., 2016). Meanwhile, a whole‐grain diet also had favorable effects on other lipid profiles. According to Giacco et al., intake of whole‐meal wheat foods for 3 weeks significantly reduced postprandial plasma LDL cholesterol in their randomized clinical study (Giacco et al., 2010). In a meta‐analysis of randomized controlled studies, whole‐grain diets led to a greater decrease in total and LDL cholesterol than nonwhole‐grain diets (Hollaender et al., 2015).

In particular, the consumption of a whole‐grain diet can be an important dietary factor among Koreans who consume rice as a staple food. Asian populations, including Korean, Japanese, and Chinese, consume white rice, which is approximately 30–40% of their total carbohydrate intake (Eshak et al., 2014; Rebello et al., 2014). Koreans derive about 60% of their energy from carbohydrate intake, while adults in the US and UK derive about 45–47% energy from carbohydrate intake (U.S. Department of Agriculture & Agricultural Research Service, 2020; Ministry of Health and Welfare, 2020; Public Health England, 2020; Song & Song, 2019). Indeed, white rice consumption was positively associated with the prevalence of dyslipidemia in Korean adults (Song & Song, 2019). In addition, among subjects following the “Korean healthy” dietary pattern, characterized by whole grains, legumes, vegetables, and fruits, the highest score group showed significantly lower TG levels than the lowest score group, while among the subjects following the “rice and vegetables” dietary pattern, consisting of white rice, vegetables, and eggs, showed no significant relationship with triglyceride levels in Korean population with type 2 diabetes (Lim et al., 2011). In summary, the whole grain dietary pattern can play a major role in preventing hypertriglyceridemia among Koreans who consume a large amount of rice‐based carbohydrate‐rich food items.

However, the mechanism underlying the negative correlation between triglyceride level and whole‐grain diet remains unclear. Nevertheless, dietary fibers in whole grain appear to reduce blood lipid levels. In particular, the viscous fibers, such as β‐glucans, a major nutrient of whole grains, including barley and oat, have shown hypocholesterolemic effects (Tosh & Bordenave, 2020). In addition, whole grain consumption may result in either lipoprotein lipase activation or synthesis of these lipoproteins and induce a decrease in postprandial TG levels (Frayn, 2002; Giacco et al., 2014). Further studies exploring the effect of whole grain and its components on TG levels are required.

To the best of our knowledge, this is the first study to reveal the inverse association between hypertriglyceridemia and the “fruit and whole‐grain” dietary pattern in a nationally representative sample of the middle‐aged and older aged Korean adult population. However, several limitations exist in this study. First, the 1‐day, 24‐h dietary recall method, which was used for dietary assessment in KNHANES, may not accurately reflect the usual dietary intake and its long‐term effect in subjects. Second, several decisions used in the factor analysis, such as the number of extracted dietary pattern groups, categorization of food items into food groups, and the rotation method, can be subjective (Martinez et al., 1998). Finally, this study was not able to examine the causal relationship between dietary pattern and hypertriglyceridemia due to the cross‐sectional study design of KNHANES. In addition, despite adjusting for confounding variables, there may have been potential confounding bias due to the observational study design of this study. These reasons may limit the possibility of generalizing the results. Therefore, additional studies with different study designs, such as clinical or prospective cohort study are required to confirm the causal relationship of the “fruit and whole‐grain” dietary pattern with hypertriglyceridemia.

## 5 CONCLUSIONS

This study among middle and older aged Korean adults found that the “fruit and whole‐grain” dietary pattern was associated with a significantly low prevalence of hypertriglyceridemia in men, whereas the effect was only marginal in women. Our findings indicate that a high proportion of fruit and whole grain in a diet may prevent hypertriglyceridemia in middle and older aged Korean adults.

## FUNDING INFORMATION

This work was supported by the National Research Foundation of Korea (NRF) grant funded by the Korea government (MEST) (No. 2020R1C1C1014286).

## CONFLICT OF INTEREST

The authors declare that they do not have any conflict of interest.

## ETHICAL APPROVAL

This study utilized a cross‐sectional and nationally representative survey, the Korea National Health and Nutrition Examination Survey (KNHANES) 2013–2018 database. The KNHANES was approved by the Institutional Review Board of The Korea Disease Control and Prevention Agency (2013‐07CON‐03‐4C, 2013‐12EXP‐03‐5C, and 2018‐01‐03‐P‐A). Written informed consent was obtained from each participant when the surveys were conducted. This study did not require additional review and approval because the KNHANES database is publicly available.

## Supporting information


Table S1

## Data Availability

Data are available in a public, open access repository. Website: https://knhanes.kdca.go.kr/knhanes/eng/index.do.
