# Supplementary material for: The “fruit and whole‐grain” pattern is associated with a low prevalence of hypertriglyceridemia among middle and older‐aged Korean adults: Using Korea National Health and Nutrition Examination Survey 2013–2018 data
Source: Food Sci Nutr. 2022 Nov 4;11(3):1201–11. doi: 10.1002/fsn3.3128 (PMC10002937; doi:10.1002/fsn3.3128)
Supplement: Supplementary file 1 — Table S1 [file FSN3-11-1201-s001.docx]

Supplementary Table 1. Major 24 food groups consumed by the middle and older aged Koreans, the 2013–2018 KNHANES

| White rice | White rice, cooked white rice, fried rice |
| --- | --- |
| Whole-grains | Oats, barley, buckwheat, millet, sorghum, Job's tears, corn |
| Flours | Flours, mixed flours, bread crumb |
| Noodles | Instant noodles, Udon, dry noodles, buckwheat noodles, Kalguksu (chopped noodles), Jjolmyeon (spicy cold chewy noodles), spaghetti, black bean sauce noodles, starch noodles |
| Bread, sandwiches, and cereal | Bread, biscuit, snacks, cake, cereals, sandwich |
| Pizza and hamburgers | Pizza, hamburger, hot dog, pork cutlet, fried potato (French-fries), chicken cutlet, fried seaweeds, instant soup |
| Potatoes | Potato, sweet potato, dried potato, artichoke, starch |
| Fruits | Orange, tangerine, strawberry, berries, lime, lemon, litchi, mango, melon, plum, fig, banana, pear, cherry, peach, apple, apricot, pomegranate, watermelon, avocado, coconut, kiwi, pineapple, papaya, grape, dragon fruit, grapefruit. |
| Vegetables | Spinach, sprouts, lettuce, broccoli, celery, cabbage, tomato, eggplant, bell pepper, chili, onion, garlic, spring onion, cactus, pak choi, kale, cucumber, lotus root, burdock, perilla leaf, radish, beet, carrot |
| Kimchi | All Kimchi (A traditional fermented food in Korean diet, such as cabbage Kimchi, radish Kimchi, cucumber Kimchi, and other vegetable Kimchi) |
| Mushrooms | Pine mushroom, shiitake, button mushroom, oyster mushroom, black mushroom, and other mushrooms |
| Legumes and soybean products | Soybean, kidney bean, mung bean, pea, cherry bean, bean curd dregs, tofus, sword bean, red beans |
| Soybean paste | Red pepper sauce, soybean sauce, fermented soybean sauce |
| Salted foods | Salted seafood, seasoned dried radish, pickled vegetables, pickled roe |
| Eggs | Eggs, quail's egg, duck egg |
| Meats | Pork, beef, chicken, and these products, duck, rabbit |
| Fish and seafood | Fishes (salmon, mackerel, cutlassfish, flatfish, snapper, cod, and croaker), dried fish, squid, octopus, crabs, clams, conch, oyster, shrimps, seaweeds, and products |
| Dairy products | Milk, yogurt, processed milk, ice cream, cheese, cream, dried milk |
| Beverages | Coffee, tea, soft drinks (coke, cider, etc.), fruit and vegetable juice (orange juice, grape juice, etc.), fruit and vegetable extracts |
| Alcoholic beverages | Soju, beer, cocktail, vodka, rum, dry gin, makgeolli, fruit liquor, whiskey, brandy, kaoliang liquor, sparkling wine, wine |
| Sugar and syrup | Sugar, syrup, jam, marmalade, honey, grain syrup |
| Nuts | Walnut, pecan, peanut, almond, pine nut, chestnut, ginkgo nut, sesame, cashew nut, macadamia, pistachio, sunflower seed, acorn, and other seeds. |
| Oils and fats | Olive oil, sesame oil, coconut oil, sunflower seed oil, grape seed oil, corn oil, beef tallow, soybean oil, lard, peanut butter, peanut oil, butter, margarine |
| Seasonings | Soy sauce, salt, vinegar, pepper, mustard, chili sauce, salad dressing, powdered seasoning, hot sauce, dried red pepper powder, curry sauce, laurel leaf, allspice, ketchup, mayonnaise |

KNHANES: Korea National Health and Nutrition Examination Survey
